# Safe and controlled technique of aortic cannulation for thoracoabdominal normothermic regional perfusion

**DOI:** 10.1016/j.xjtc.2024.03.005

**Published:** 2024-04-27

**Authors:** Mohamed Abdullah, Ahmed Sedeek, Derek Serna-Gallegos, Danny Chu, Pyongsoo Yoon, Johannes Bonatti, Ibrahim Sultan, David J. Kaczorowski

**Affiliations:** a Department of Cardiothoracic Surgery, University of Pittsburg Medical Center, Pittsburgh, Pa; b Department of Cardiothoracic Surgery, Cairo University, Cairo, Egypt; c Veterans Affairs Pittsburgh Healthcare System, Pittsburgh, Pa

**Figure:**
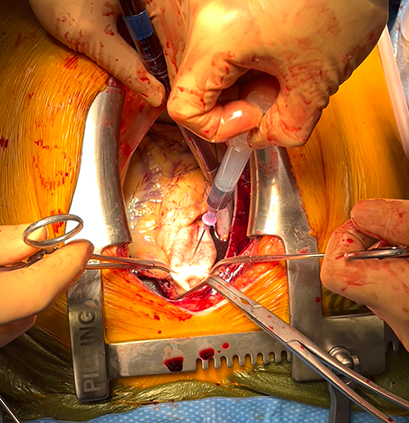
Cannulation of depressurized aorta for NRP over the wire minimizes the risk of injury.

**Central Message**

Cannulation of a depressurized aorta carries significant risk of injury to the aorta and surrounding structures. We describe an effective technique to minimize this risk.

## Case Video Summary

Video 1 highlights an efficient and safe approach to cannulate a depressurized aorta in cases of organ donation after cardiac death using thoracoabdominal normothermic regional perfusion. Institutional review board approval was not required; informed written consent was obtained from the donors’ families. After declaration of death by an independent physician, and after a standoff period has elapsed, the procedure is initiated by rapid sternotomy, opening of the pericardium, and clamping of the aortic arch vessels to prevent reperfusion of the brain. A dual-stage venous cannula is introduced into the right atrium and venous drainage is initiated to alleviate venous congestion of the organs. Next, the depressurized aorta is grasped and cannulation is performed using the Seldinger technique, reducing the risk of complications such as aortic dissection or airway injury. A miniaturized cardiopulmonary bypass circuit is used for this approach. Video 1 outlines the steps, instruments, and techniques utilized to ensure a safe and effective procedure.

## Conclusions

After cardiac arrest, the aorta becomes depressurized, and its cannulation for initiation of cardiopulmonary bypass may be challenging, carrying the risk of injuring the aortic wall, which could result in hematoma formation, disruption of the wall, or dissection. There is also risk of injury to the neighboring structures, including the major airways, which would compromise the quality of the procured lungs for transplantation. Our described technique for cannulating the depressurized aorta for thoracoabdominal normothermic regional perfusion during donation after cardiac death organ procurement minimizes this risk. Video 1 demonstrates the rapid resumption of myocardial activity and robust cardiac function, as confirmed by transesophageal echocardiography, which also confirms the absence of aortic dissection. This approach offers a potential method for optimal utilization of the NRP method to expand the pool of organ donors, addressing a critical need in the field of organ transplantation.

### Webcast

You can watch a Webcast of this AATS meeting presentation by going to: https://www.aats.org/resources/safe-and-controlled-technique-7032.

**Figure:**
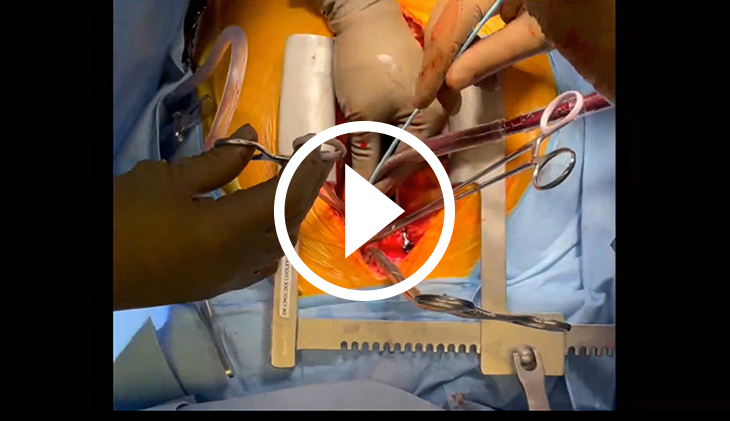

## Conflict of Interest Statement

The authors reported no conflicts of interest.

The *Journal* policy requires editors and reviewers to disclose conflicts of interest and to decline handling manuscripts for which they may have a conflict of interest. The editors and reviewers of this article have no conflicts of interest.

## Supplementary Data

Video 1 The video demonstrates the expeditious chest entry and cannulation for cardiopulmonary bypass in cases of donation after cardiac death, highlighting the technique for aortic cannulation. Video available at: https://www.jtcvs.org/article/S2666-2507(24)00133-0/fulltext.

