# Supplementary figures and images for: Safe and controlled technique of aortic cannulation for thoracoabdominal normothermic regional perfusion
Source: JTCVS Tech. 2024 Apr 27;25:33–4. doi: 10.1016/j.xjtc.2024.03.005 (PMC11184591; doi:10.1016/j.xjtc.2024.03.005)

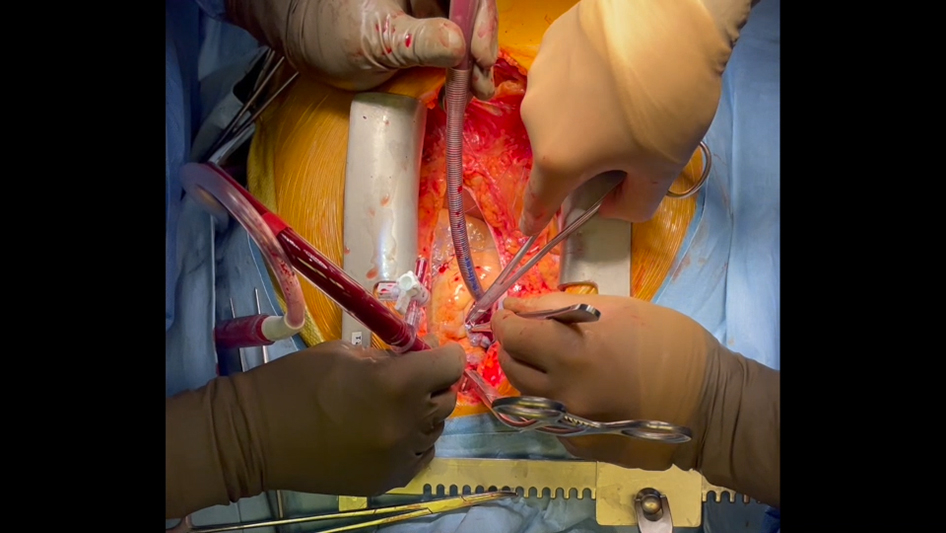

Supplement: Video 1 — The video demonstrates the expeditious chest entry and cannulation for cardiopulmonary bypass in cases of donation after cardiac death, highlighting the technique for aortic cannulation. Video available at: https://www.jtcvs.org/article/S2666-2507(24)00133-0/fulltext. [file fx2.jpg]
